# TECHNOLOGY FOR SUPPORTING HEALTH AND WELLNESS

**DOI:** 10.1093/geroni/igae098.1538

**Published:** 2024-12-31

**Authors:** Walter Boot, Xin Yao Lin

**Affiliations:** Chair; Co-Chair

## Abstract

As the population ages, there is a growing need for technology to support the health and wellbeing of older people. However, technology solutions, for them to be successful, need to recognize the needs, preferences, and abilities of older people, and must be evaluated rigorously for efficacy. This talk provides an overview of the potential and pitfalls of using technology to support health and wellness. Hye Soo Lee, PhD, explores the role of technology in supporting Medicare-related decision-making by older adults, highlighting the need for improved training and the consideration of user characteristics to enhance engagement with government-provided digital tools. Paul Freddolino, PhD, MDiv, presents findings from telehealth training sessions with older adults, emphasizing the importance of end-user feedback in refining patient-focused content and increasing patient engagement in healthcare. Justin Lam, BS, discusses the design of age-friendly web interfaces, focusing on optimizing user interaction patterns and enhancing readability to accommodate the nuanced needs of older adults and their care partners. Elena Remillard, M.S., shares insights from deploying ‘Telewellness’ programs, such as Tele Tai Chi, in community settings, identifying barriers and facilitators to their delivery and discussing the potential of different models to increase access to exercise classes for older adults with mobility disabilities. Leo He, BSc, introduces a novel, radio-based, non-contact system for monitoring nighttime vital signs and sleep, offering a passive, cost-effective solution for home-based health monitoring that avoids the physical and cognitive burdens associated with existing technologies, aiming to improve health intervention and quality of life for older adults.

